# Diabetic foot ulcer healing with polylactic acid membrane assessed by thermographic imaging: a case report

**DOI:** 10.3389/fmed.2025.1568144

**Published:** 2025-07-09

**Authors:** Víctor Manuel Loza-González, Mario Aurelio Martínez-Jiménez, Ana Lorena Novoa-Moreno, José Luis Ramírez-GarcíaLuna, Alejandra Ortiz-Dosal, Eleazar Samuel Kolosovas-Machuca

**Affiliations:** ^1^Doctorado Institucional en Ingeniería y Ciencia de Materiales, Universidad Autónoma de San Luis Potosí, San Luis Potosí, Mexico; ^2^Burn Unit, Hospital Regional de Alta Especialidad Dr. Ignacio Morones Prieto, San Luis Potosí, Mexico; ^3^Division of Experimental Surgery, Faculty of Medicine, McGill University, Montreal, QC, Canada; ^4^Faculty of Sciences, Universidad Autónoma de San Luis Potosí, San Luis Potosí, Mexico; ^5^Investigadora por Mexico, Programa Cátedras, Secretaría de Ciencia, Humanidades, Tecnología e Innovación (SECIHTI), Ciudad de Mexico, Mexico; ^6^Coordinación para la Innovación y Aplicación de la ciencia y Tecnología, Universidad Autónoma de San Luis Potosí, San Luis Potosí, Mexico

**Keywords:** infrared thermography, diabetic foot, polylactic acid membrane, ulcer healing, diabetes mellitus

## Abstract

Diabetic foot ulcers reduce patient’s quality of life and increase treatment costs, evaluating this condition is promising. We report the clinical progression of an elderly patient who developed a diabetic foot ulcer following minor trauma. She was treated for over 2 months until her condition progressed to Grade 4 on the Wagner Classification of Diabetic Foot. A Serial infrared thermography was performed. The treatment plan included antibiotics and a polylactic acid membrane until complete ulcer remission. Thermographic imaging showed a quantitative reduction of the temperature differences between the affected and contralateral foot from an initial ΔT: −4.6°C ± 2.4, progressing to ΔT: −1.7°C ± 1.6 at 12 weeks, and a temperature reduction from the proximal third tibial area with ΔT: −1.7°C ± 0.6, with a change to ΔT 0.8°C ± 0.4 at 12 weeks near the metatarsal bone region. This article underscores the use of infrared thermography to give physicians feedback on ulcer healing progression phases and treatment effectiveness.

## Introduction

Approximately 415 million people worldwide are affected by diabetes, and between 4% and 10% will develop diabetes-related foot ulcers (DFUs) (1). These wounds are among the most severe complications of diabetes, as their occurrence precedes over 80% of lower extremity amputations and increase mortality and economic burdens (2). Most DFUs appear in patients with peripheral neuropathy and vascular disorders. Regions at risk of developing DFUs can easily be identified through changes in the local skin temperature when comparing both feet (3). For example, in a recent meta-analysis, the authors found that daily at-home foot temperature monitoring and reduction of ambulatory activity in response to “hotspots,” defined as temperatures over > 2°C in the affected foot vs. non-affected foot, significantly reduce the risk of developing ulceration amongst moderate or high-risk patients (4). Therefore, if foot temperature changes are detected early during the disease, early interventions can be started to prevent the ulcer from developing (5). Moreover, if and once the ulcer develops, heat assessment can be used to establish a treatment plan to identify healing vs. non-healing ulcers, provide adequate follow-up, and adjust interventions accordingly to the patient’s evolution to avoid amputations (1).

In our facility, wound assessment is initially performed by physical examination, which is considered the standard of care worldwide (6). Physical examination allows the medical team to diagnose the status of the patient and make decisions based on peripheral skin color, wound bed characteristics, humidity levels, and exudate, among other factors. However, this examination is subjective and prone to error. Infrared thermography (IRT) can provide an additional quantitative measurement of the temperature of a wound related to tissue damage, complementing the clinical inspection (7). The value of IRT for clinical assessment of diabetic wounds mainly lies in the fact that vascular and perfusion alterations lead to temperature fluctuations; therefore, the patient’s evolution can be objectively monitored through the disease course (8, 9).

Thermographic imaging allows the creation of thermal maps of scanned areas, which provide immediate qualitative and quantitative data. Modern thermal cameras are portable, have high resolution, and are available at low cost (10). Their use in the human body has allowed healthcare practitioners over the past few decades to evaluate ulcers, diabetic wounds, and burns, as well as to perform preoperative planning of reconstructive surgery, (2, 7, 8, 10–12).

Cellular, acellular, and matrix-like products (CAMPs) are essential tools for the management of hard-to-heal wounds, providing structural support and biochemical signaling to promote tissue regeneration. Traditional CAMPs include allografts, xenografts, and biologically derived matrices, which have demonstrated efficacy in improving wound healing outcomes. However, these biologic-based products pose limitations, including batch-to-batch variability, potential immunogenicity, high costs, and logistical challenges associated with storage and handling. These factors have driven interest in alloplastic (synthetic) alternatives, which offer standardized composition, controlled degradation kinetics, and improved biocompatibility (13).

Among alloplastic CAMPS, poly-lactic acid (PLA) wound closure matrices represent a promising option for the treatment of DFUs. These matrices initially serve as a scaffold, integrating with the wound bed to provide mechanical support. However, upon degradation, they release lactate, a key signaling molecule that has been shown to stimulate angiogenesis, regulate inflammation, and promote ECM remodeling (14–17). These bio-inductive properties directly address the underlying pathophysiology of DFUs, where inadequate vascularization and prolonged inflammation remain major barriers to healing. Furthermore, their ability to resist infection, modulate the wound microenvironment, and facilitate sustained tissue remodeling makes them particularly well-suited for hard-to-heal diabetic wounds, including those in resource-limited settings where access to biological grafts is constrained. As such, the use of PLA matrices in DFUs has demonstrated accelerated healing times and superior wound closure rates compared to collagen-based matrices and xenografts, positioning them as a viable alternative to traditional biologic CAMPs (18, 19).

Given the interplay between vascularization, inflammation, and extracellular matrix remodeling in DFU healing, infrared thermography (IRT) may provide valuable and real-time insights into the wound healing trajectory following the application of bio-inductive therapies like PLA wound closure matrices. As lactate release from PLA degradation is known to enhance angiogenesis and modulate inflammation, thermographic imaging allows for real-time visualization of perfusion changes, which serve as objective indicators of tissue viability and regenerative activity. Additionally, thermal pattern analysis may help predict early wound closure potential, identify areas at risk of delayed healing, and assess treatment response longitudinally, complementing standard clinical evaluations. In this case report, we explore the application of IRT as a functional imaging tool to monitor the progressive vascular and metabolic changes induced by PLA matrices, providing objective evidence of their impact on wound repair dynamics in a diabetic foot ulcer.

## Clinical case

A 67 years-old female patient with a 13 years history of insulin-dependent type 2 diabetes and pancreatic insufficiency, presented with a 2 months-old Wagner grade 4 diabetic foot ulcer (DFU) affecting the left first toe, characterized by significant necrosis, peri-wound maceration, and signs of deep tissue involvement. The ulcer developed following a fall at home, after which the patient initially received standard wound care and systemic antibiotic therapy (amoxicillin/clavulanic acid) at another facility. Despite these interventions, the wound failed to improve, and she was referred to our center for advanced wound management.

At her initial evaluation, the wound exhibited significant necrosis, erythema and discharge prompting extensive surgical debridement Osteomyelitis was ruled out through a negative probe-to-bone test during the patient’s initial evaluation. Management included standard offloading measures and continuation of the antibiotic therapy for one more week, completing a total of 10 days, based on the patient’s clinical response. Afterward, following infection resolution and confirmation of a well-prepared wound bed, therapy with a PLA-guided wound closure matrix was initiated. The matrix was applied weekly for three consecutive weeks, according to the humidity and integrity of the previously placed membrane. No difficulties in adherence were reported, and the patient did not report any discomfort or adverse reactions after application. At each dressing change, granulation tissue formation was observed, with progressive wound contraction and tissue remodeling observed over the course of treatment. Additionally, the patient maintained adequate glycemic control during the treatment period, with blood glucose levels ranging from 101 to 130 mg/dL and HbA1c of 6.4%. The patient continued with weekly follow-up visits until healing.

During every visit, IRT imaging was performed using a Flir T600 infrared camera (FLIR System, Wilsonville, OR) in a controlled environment with a temperature of 22°C, 40%, atmospheric humidity, and standardized light conditions. Scans were performed from a distance of 0.5 m. Before each scan, dressings were removed, and the wound was cleaned and dried with sterile gauze. The patient remained with the affected extremities uncovered for 20 min before scanning to avoid measurement errors. All imaging was done following the TISEM checklist and the Glamorgan protocol (20, 21).

The regions of interest for IRT analysis were the toes and tibial areas of the affected and healthy contralateral limbs. The analysis was performed using the FLIR Ignite Sync software (FLIR Systems, version 1.5.0.2169) by a researcher blinded to the clinical data and final diagnosis. The skin emissivity was set at 0.98, for all acquisitions, delimiting the regions of interest in the affected extremity. The differences in average temperatures (ΔT) of the areas of interest between the affected and contralateral limbs were calculated.

**FIGURE 1:**
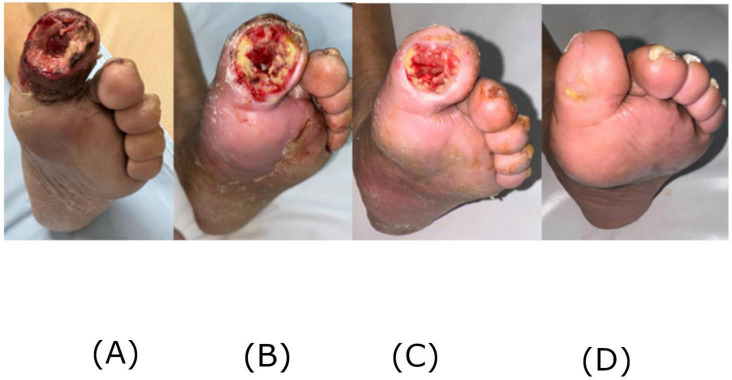
Clinical photographs of the ulcer remission from left to right at the initial consultation **(A)**, at 3 weeks **(B)**, 9 weeks **(C)**, and 12 weeks **(D)**.

**FIGURE 2:**
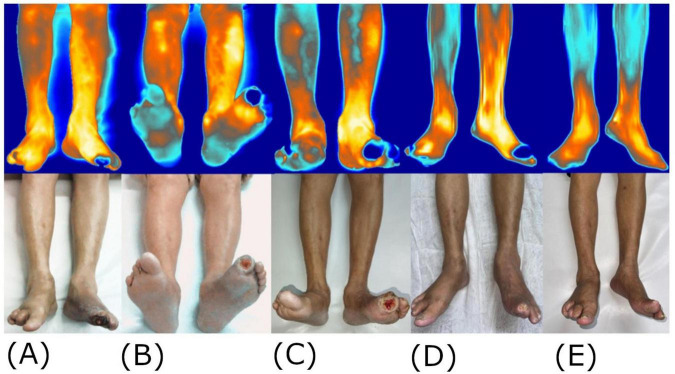
Use of thermography for ulcer remission assessment. Frontal areas with the lowest temperature are displayed in blue and the highest temperature in white. Change progressions are depicted until similar views were obtained after the wound complete healing, from left to right, at the initial consultation **(A)**, at 3 weeks **(B)**, 6 weeks **(C)**, 9 weeks **(D)**, and 12 weeks **(E)**.

**FIGURE 3:**
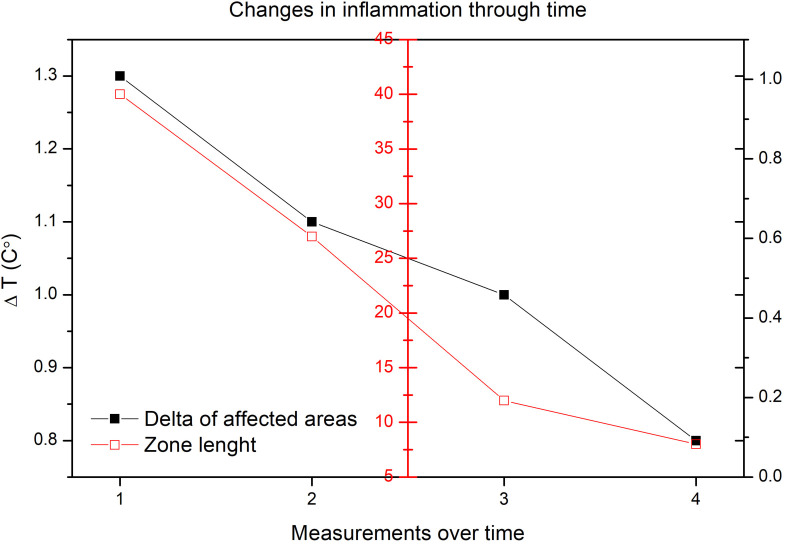
Change in the ΔT values through time between the toes in the affected limb and the contralateral limb (black). Change of zone length of the affected limb with the biggest ΔT values in comparison with the contralateral limb (red).

The ΔT between the affected and contralateral limbs was registered until healing, and reference clinical photographs were taken (Figures 1, 2). A thermal change was observed with a decrement to 0.3°C in the negative ΔT measurements between the area of interest and the reference area of the toes (Supplementary Table 1). To register IRT changes, a graph of ΔT register was created. A graph of the lesion length reduction was included (Figure 3 and Supplementary Figure 1).

Full closure of the wound was achieved at 12 weeks. However, follow-up was continued for one more month until equalization of the ΔT. Over the ensuing weeks, the thermal progression was visualized with a tendency to 0°C in the positive ΔT values observed between the peripheral tibial areas of the affected limb and the contralateral limb. Also, a reduction in the length of the affected area was measured until the affected limb exhibited a localized increase at the metatarsal bone area (Table 1).

**TABLE 1:** Change in the Δt values through time course (weeks) between peripheral wound areas with bigger registered Δt values (left columns).

Weeks of wound healing progression	Delta	Limit of inflammation	Length (cm)
**Change of thermal patterns through time**			
0	1.3	The proximal third of the leg	40
3	1.1	The middle third of the leg	27
6	1	The distal third of the leg	12
9	0.8	The metatarsal area	8
12	0.8	The metatarsal area	8

Length reduction of the affected peripheral wound area from the knee to the metatarsal bone area (right columns).

## Discussion

In this case report, infrared thermography (IRT) effectively captured the dynamic changes associated with ulcer healing, providing real-time visualization of the temperature redistribution associated with perfusion, inflammation, and tissue remodeling. The ulcer size decreased simultaneously with the temperature, consistent with other studies (22). The thermal data allowed for an objective, quantitative assessment of the ulcer’s progression through distinct healing phases, which are often difficult to assess through standard clinical inspection alone.

Infrared thermography takes advantage of temperature changes caused by diseases that alter local circulation. The decreased temperature observed in the patient’s affected limb may be attributed to vascular insufficiency, which leads to reduced blood flow and, consequently, a lower temperature in the affected area (23). Infrared thermography has been previously used in patients with peripheral artery disease for screening purposes, identifying areas of impaired perfusion, and measuring the effects of medical treatment (24).

Throughout the treatment period, we identified three distinct IRT-defined healing phases: (1) an inflammatory phase, characterized by widespread thermal asymmetry (ΔT > 2°C) extending from the ulcer to the proximal third of the tibia, likely representing inflammatory hyperemia and possible residual infection; (2) a proliferative phase, during which the extent of hyperthermic zones decreased, and ΔT gradually reduced to values below 1°C, suggesting resolution of acute inflammation and increased angiogenic activity; and (3) a remodeling phase, where ΔT values fully normalized (ΔT = 0°C), correlating with initial epithelial closure and subsequent tissue stabilization. These findings align with previous literature demonstrating that progressive thermal equalization with adjacent uninjured skin is a hallmark of ulcer resolution (25, 26).

The observed temperature patterns provide valuable physiological insights into the mechanisms by which PLA-guided wound closure matrices support DFU healing. During the proliferative phase, lactate release from matrix degradation is known to stimulate hypoxia-inducible factor-1α (HIF-1α) pathways, leading to vascular endothelial growth factor (VEGF) upregulation and neovascularization (17). These angiogenic effects are crucial in DFU healing, as chronic wounds are often characterized by hypoxia, impaired microcirculation, and insufficient growth factor production. The thermal data confirmed this effect, as increasing tissue perfusion correlated with progressive ΔT reduction and wound closure. Notably, IRT imaging allowed for early detection of angiogenic responses, well before visible clinical improvement was observed.

Importantly, thermographic assessment also serves as a potential predictive tool for guiding clinical interventions (24). In this case, the initial thermal signature (ΔT > 2°C) suggested an underlying inflammatory response, consistent with previous studies showing that wounds with a high ΔT have a high probability of inflammation or infection (27, 28). This early identification allowed for closed monitoring, potentially preventing further complications. Conversely, persistent inflammatory hyperthermia or failure to reach ΔT normalization could serve as an early warning sign of impaired healing, enabling timely clinical intervention.

Given its non-invasive nature, ease of use, and ability to provide real-time quantitative data, IRT has strong potential to become a standard adjunctive tool in DFU management. Previous work has evaluated the use of this technology in diabetic foot, establishing infrared thermography as a well-recognized risk assessment tool for diabetic foot ulcers. It can serve as a complementary method for the timely identification of pre-ulcerative signs and is particularly useful for detecting problematic areas that may go unnoticed due to sensory neuropathy (2). Modern thermal cameras are highly portable, cost-effective, and user-friendly, allowing for bedside monitoring and remote telemedicine applications (10, 12, 29). Moreover, the integration of IRT-based predictive models could enable clinicians to forecast healing trajectories, optimize treatment plans, and implement early therapeutic adjustments (7).

As limitations, this article includes clinical data and thermal scans from a single patient. Image acquisition was performed according to the availability within the medical consultation schedule. There is currently no consensus regarding the optimal timing for thermographic imaging in clinical settings. Additionally, anatomical changes are common in some patients with diabetic foot, particularly in those who develop Charcot neuroarthropathy. Alterations in the arch and angulation of the foot can affect infrared thermography readings if not properly considered, as the emissivity and apparent temperature of an object may vary depending on the viewing angle. Moreover, thermographic imaging should be performed in controlled, clean environments to minimize the risk of measurement alterations and to ensure patient safety by avoiding potential wound contamination (7).

Incorporating infrared thermography into standard DFU monitoring protocols would enhance clinical decision-making, facilitate early detection of complications, and provide objective measures of treatment efficacy. Given the strong correlation between temperature dynamics and wound healing physiology, we propose that IRT should be routinely employed in the assessment of DFUs, particularly when utilizing bio-inductive technologies such as PLA-guided wound closure matrices. Future research should focus on establishing standardized ΔT thresholds for different DFU severities, validating its use in predictive healing models, and further exploring its role in personalized wound care strategies.

## Conclusion

Infrared thermography presents a valuable, non-invasive tool for the initial assessment, longitudinal monitoring, and treatment evaluation of DFUs. By quantifying thermal patterns in both the ulcer bed and surrounding tissue, IRT provides objective, real-time data that complements standard clinical assessments. The ability to track perfusion changes, detect prolonged inflammation, and predict healing trajectories offers a significant advantage in optimizing wound management strategies.

Integrating IRT-based analytics into DFU care could enable early identification of delayed healing, guide therapeutic adjustments, and facilitate personalized treatment approaches. Furthermore, advances in thermal imaging technology and artificial intelligence-driven pattern recognition hold promise for the development of predictive models that can forecast healing timelines and intervention outcomes. As the field progresses, standardizing IRT-based diagnostic criteria and incorporating this technology into routine clinical practice could significantly improve DFU management, reduce complications, and enhance patient outcomes.

## Data Availability

The original contributions presented in this study are included in this article/Supplementary material, further inquiries can be directed to the corresponding author.
