# Supplementary figures and images for: Diabetic foot ulcer healing with polylactic acid membrane assessed by thermographic imaging: a case report
Source: Front Med (Lausanne). 2025 Jul 9;12:1568144. doi: 10.3389/fmed.2025.1568144 (PMC12283997; doi:10.3389/fmed.2025.1568144)

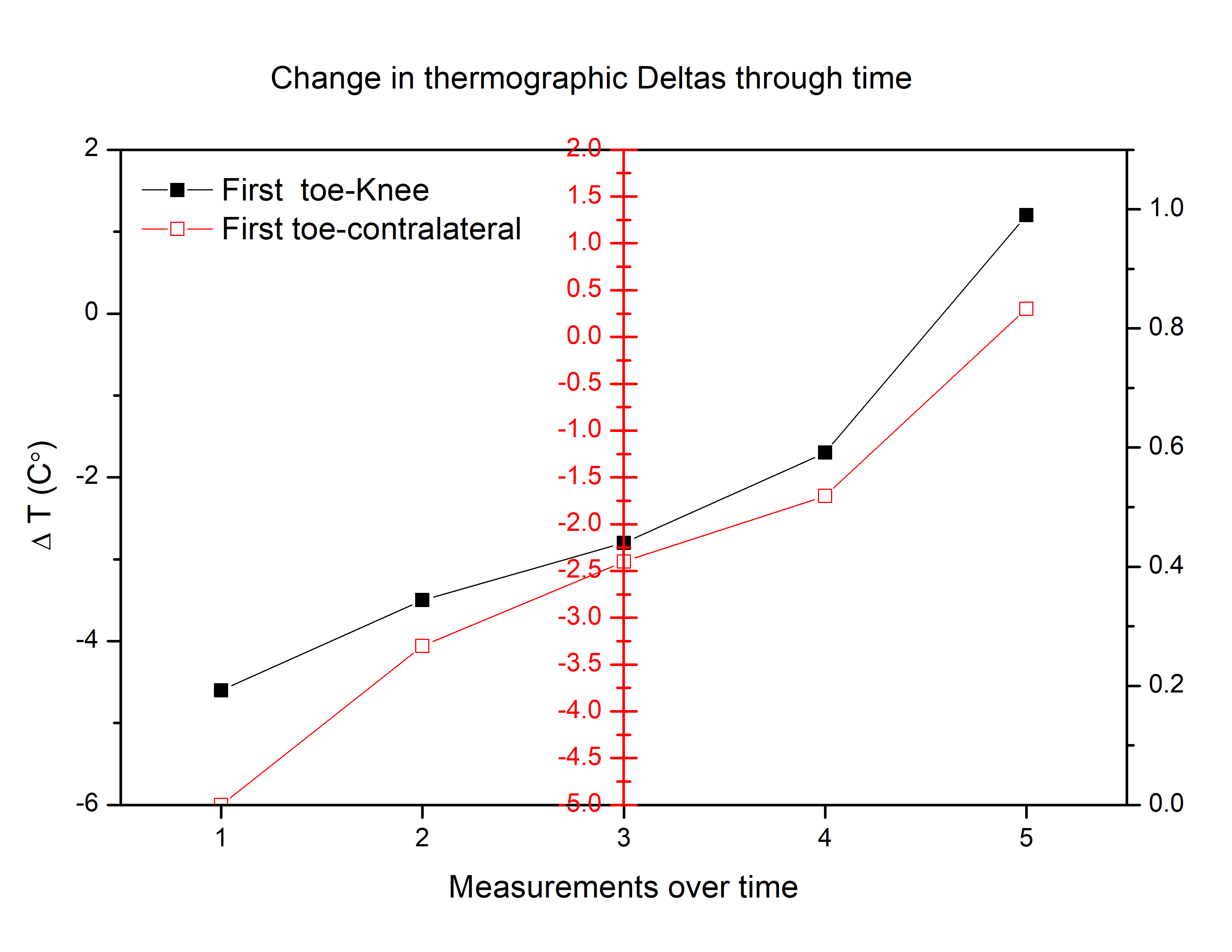

Supplement: Supplementary file 1 [file Image_1.jpeg]
