# Supplementary material for: Diabetic foot ulcer healing with polylactic acid membrane assessed by thermographic imaging: a case report
Source: Front Med (Lausanne). 2025 Jul 9;12:1568144. doi: 10.3389/fmed.2025.1568144 (PMC12283997; doi:10.3389/fmed.2025.1568144)
Supplement: Supplementary file 2 [file Table_1.docx]

**Supplementary Table 1**. The middle column depicts the changes in the Δt values through time between the affected left knee and the first left toe. The right column depicts the changes in the Δt values of the affected and the normal toe.

| **Change of thermal patterns through time** | | |
| --- | --- | --- |
| **Weeks of wound healing progression** | **Delta between first left toe and left knee** | **Delta between first left toe and first right toe** |
| 0 | -4.6 | -5.2 |
| 3 | -3.5 | -3.3 |
| 6 | -2.8 | -2.6 |
| 9 | -1.7 | -1.7 |
| 12 | 1.2 | 0.3 |
